# Non‐neuronal, but atropine‐sensitive ileal contractile responses to short‐chain fatty acids: age‐dependent desensitization and restoration under inflammatory conditions in mice

**DOI:** 10.14814/phy2.12759

**Published:** 2016-04-06

**Authors:** Masako Yajima, Shunsuke Kimura, Shinichiro Karaki, Junko Nio‐Kobayashi, Takeshi Tsuruta, Atsukazu Kuwahara, Takaji Yajima, Toshihiko Iwanaga

**Affiliations:** ^1^ Laboratory of Histology and CytologyGraduate School of MedicineHokkaido UniversitySapporoJapan; ^2^ Laboratory of PhysiologySchool of Food and Nutritional SciencesGraduate Division of Nutritional and Environmental SciencesUniversity of ShizuokaShizuokaJapan; ^3^ Meiji Dairies Research ChairCreative Research InstitutionHokkaido UniversitySapporoJapan; ^4^ Department of Animal ScienceGraduate School of Natural Science and TechnologyOkayama UniversityOkayamaJapan

**Keywords:** Acetylcholinesterase, age, ileal contraction, inflammatory condition, non‐neuronal acetylcholine, short‐chain fatty acids

## Abstract

Intestinal epithelial cells sense short‐chain fatty acids (SCFAs) to secrete non‐neuronal acetylcholine (ACh). However, the roles of luminal SCFAs and epithelial ACh under normal and pathological conditions remain unknown. We examined ileal contractile responses to SCFAs at different ages and their mucosal cholinergic alterations under inflammatory conditions. Ileal contractile responses to SCFAs in 1‐day‐old pups to 7‐week‐old mice were compared using an isotonic transducer, and responses to an intraperitoneal injection of lipopolysaccharide (LPS) were analyzed in 7‐week‐old mice. The mRNA expression levels of a SCFA activate free fatty acid receptor, acetylcholinesterase (AChE), choline acetyltransferase (Chat), and choline transporter‐like protein 4 (CTL4) were measured using real‐time quantitative RT‐PCR. AChE was analyzed by histochemical and optical enzymatic assays. Atropine‐sensitive ileal contractile responses to SCFAs occurred in all 1‐day‐old pups, but were frequently desensitized after the weaning period. These contractile responses were not inhibited by tetrodotoxin and did not appear when the mucosal layer had been scraped off. Contractile desensitization in 7‐week‐old mice was abolished in the presence of the AChE inhibitor, eserine, which was consistent with increased AChE activity after weaning. Ileal contractions to SCFAs in adult mice were restored by LPS, which significantly increased the epithelial mRNA expression of Chat and CTL4. Atropine‐sensitive ileal contractile responses to SCFAs constitutively occur in the newborn period, and are desensitized during developmental stages following the up‐regulated expression of AChE in the villous mucosa, but are restored under inflammatory conditions possibly via the release of epithelial ACh.

## Introduction

Short‐chain fatty acids (SCFAs) are produced by bacterial fermentation, accumulate in the lumen of the gastrointestinal tract after birth, and their amounts and compositions are altered by colonized bacterial species. SCFAs have been shown to evoke diversified effects on the host. They act not only as energy sources (Livesey and Elias 1995) but also as effectors of body energy utilization through the activation of SCFA activate free fatty acid receptor FFA2 (GPR43), which suppresses insulin‐mediated fat accumulation in adipocytes (Kimura et al. 2013). SCFAs were previously reported to have direct functions in the intestinal mucosa: chemical stimulators of mesenteric vasodilatation (Knock et al. 2002), colonic contractions (Yajima 1985; Mitsui et al. 2005), peristalsis (Grider and Piland 2007), mucin secretion (Sakata and Setoyama 1995), and chloride secretion (Yajima 1988). Furthermore, recent studies demonstrated that SCFAs modulate immune responses (Sina et al. 2009; Maa et al. 2010; Vinolo et al. 2011) as well as the production of chemokines and cytokines in intestinal epithelial cells via the SCFA receptors, GPR41 and GPR43 (Kim et al. 2013). SCFAs have also been shown to exert protective effects against intestinal inflammation via SCFA receptors in mice (Sina et al. 2009; Kim et al. 2013). The simultaneous luminal infusion of acetate and dextran sulfate sodium into germ‐free mice also had anti‐inflammatory effects (Maslowski et al. 2009). However, developmental changes in SCFA responses or expression profiles and the involvement of intestinal‐epithelial SCFA receptors in systemic inflammation currently remain unclear.

Acetylcholine (ACh) is a well‐known neurotransmitter and is involved in non‐neuronal cholinergic systems in health and disease (Beckmann and Lips 2013); its production has been detected in many non‐neuronal cells such as keratinocytes (Grando et al. 1993), the respiratory epithelium (Reinheimer et al. 1998), and cardiomyocytes (Rana et al. 2010). In the digestive tract, the epithelial cells of the lower small intestine in mice possess choline acetyltransferase (Chat) (Gautron et al. 2013; Takahashi et al. 2014). We recently reported that the luminal sensing of SCFAs evoked the release of ACh from colonic epithelial cells to the serosal side, and eventually induced the secretion of chloride in the rat colon (Yajima et al. 2010). ACh is also known as an active ligand that causes intestinal contractions similar to SCFAs in neonatal rats (Singh and Mandal 2013). Although the regulatory mechanisms of the non‐neuronal cholinergic system currently remain unclear, ACh and SCFAs may coincidentally play a role in the regulation of intestinal contractile responses via the sensing of SCFAs. Therefore, we herein attempted to elucidate the involvement of epithelial ACh in ileal contractile responses to SCFAs during developing stages with or without systemic inflammation in mice.

## Materials and Methods

### Animals

This study was approved by the Hokkaido University Animal Committee; all animals were maintained in accordance with the Hokkaido University guidelines for the care and use of laboratory animals. Seven pregnant time‐dated specific pathogen‐free BALB/cCrSlc mice were purchased from Sankyo‐Labo‐Service Co., Ltd. (Tokyo, Japan). Pregnant animals were housed individually under environmentally controlled temperature (25 ± 2°C), humidity (50 ± 5% humidity), and light (12‐h light–dark cycle) conditions. They had free access to water and feed (CA‐1; CLEA Japan, Tokyo, Japan) and were allowed to deliver spontaneously. Pups were weaned 21 days after birth as 0 day of the birth day, and were housed separately by sex and siblings until they reached 7 weeks old. Male pups were used at 7 weeks of age in order to avoid variations due to menstrual cycles. Six‐week‐old specific pathogen‐free male mice were additionally purchased from Sankyo‐Labo‐Service Co., Ltd., and housed as five animals per cage in order to accustom them under the same conditions for 1 week until they reached 7 weeks of age.

### Preparation of ileal segments

Mice were asphyxiated with CO_2_ gas and were immediately exsanguinated after decapitation. Ileal segments were immediately removed as three portions; one portion was examined immediately for ileal contractile responses to SCFAs, while the other two were used in analyses of mRNAs and AChE activities. The villous mucosa was isolated by cutting with fine scissors to exclude the myenteric and submucous nerve plexuses, and the villous epithelium was isolated using the method described by Hase et al. (2005) with modifications. Briefly, small pieces of the ileum were soaked in ice‐cold Hank's balanced salt solution (Life Technologies, Grand Island, NY) containing 30 mmol L^−1^ EDTA and 5 mmol L^−1^ dithiothreitol. After being incubated with gentle shaking for 20 min on ice, the epithelial monolayer was carefully separated from the lamina propria of the mucosa by manipulation with a fine needle under stereomicroscopic monitoring. The villous mucosa and villous epithelium were stored at −80°C until later use. All samples for mRNA measurements were stored after immediately dipping in RNA *later* (QIAGEN, Tokyo, Japan) followed by overnight refrigeration.

In the histochemical enzyme assay, mice, except for 1‐day‐old pups, were perfused with fixative solution (4% paraformaldehyde in 0.1 mol L^−1^ phosphate buffer, pH 7.3) after flushing blood out by saline through the cardiac ventricle under deep anesthesia using pentobarbital chloride. One‐day‐old pups were asphyxiated with CO_2_ gas and immediately exsanguinated following bloodletting. Ileal segments were immediately removed on ice into fixative solution.

### Measurement of ileal contractions

As described above, ileal segments were gently isolated in order to avoid any pulling of the tissues and placed in warm Krebs bicarbonate saline solution containing (in mmol L^−1^): NaCl: 119; CaCl_2_: 1.25; MgCl_2_: 1; K_2_HPO_4_: 2.2; KH_2_PO_4_: 0.2; NaHCO_3_: 21, and glucose: 10. The solution was bubbled with a gas mixture of 95% O_2_ and 5% CO_2_ (pH 7.4, 37°C). A segment was then tied with a polyester thread at both ends at a distance of 0.8 cm. The luminal contents were gently washed out by cutting longitudinally. The segments were perpendicularly aligned gently in the Magnus isotonic‐transducing system; a 20‐mL bath (Magnus glass‐chamber: IWASHIYA KISHIMOTO MEDICAL INSTRUMENT, Kyoto, Japan) and isotonic transducer (TD‐112S & JD‐112S: Nihon Kohden, Tokyo, Japan), in order to avoid any pulling and were warmed with circulating water at 37°C. The contractile current was recorded as 1 mm/2 mV by the Power Lab system (AD Instruments, Bella Vista, Australia). Contractile responses to SCFAs were detected as longitudinal contractions of the terminal ileum.

After changing Krebs solution in the Magnus chamber three times, tissues were stabilized for approximately 40 min prior to testing contractile activity with acetate, propionate, or inhibitory drugs. Sodium acetate (1 mol L^−1^) and sodium propionate (1 mol L^−1^) (Kanto Chemical Co., Inc., Tokyo, Japan), 20–200 *μ*L, were added to the Magnus chamber, which was filled with 20 mL of Krebs solution and bubbled with a gaseous mixture of 95% O_2_ and 5% CO_2_ (pH 7.4, 37°C). Appropriate concentrations of other drugs including atropine sulfate salt monohydrate (SIGMA, St. Louis) as an inhibitor of muscarinic receptors for ACh, acetylcholine chloride (SIGMA, St. Louis) as an agonist for contractions, tetrodotoxin (SIGMA, St Louis) as an neurotoxin and/or eserine hemisulfate salt (SIGMA, St. Louis) as an inhibitor of AChE, were added at a volume of 10 and 20 *μ*L, respectively. Washing procedures were performed inside the Magnus chamber in order to avoid the mixing of these reagents.

### Injection of lipopolysaccharide (LPS)

Systemic inflammation was induced in 7‐week‐old mice by a peritoneal injection of LPS (*E. coli* 055:B5, SIGMA L2880) at a concentration of 0.05 mg kg^−1^ body weight overnight prior to testing contractile activity.

### Assay of acetylcholinesterase (AChE)activity

In the histochemical enzyme assay: Ileal tissues were postfixed in the same fixative at 5°C for 6 h and then placed in 0.1 mol L^−1^ phosphate buffer containing 30% sucrose in 0.1 mol L^−1^ phosphate buffer (pH 7.3) at 5°C for at least 16 h until embedding in OCT compound and freezing. The tissue was sectioned in 10–12‐*μ*m thick slices at −15°C using a cryostat (LEICA CM3050, Wetzlar, Germany). The staining procedure was a modified method of Karnovsky and Roots by Tago et al. (1986). Sections on a glass slide were placed in 0.1 mol L^−1^ maleate buffer (pH 6.0) for 5 min. In order to induce the AChE enzymatic reaction, the slide was incubated in assay medium (0.17 mmol L^−1^ acetylthiocholine iodide, 0.5 mmol L^−1^ sodium citrate 0.1 mol L^−1^ PB (pH 6.0), 3 mmol L^−1^ CuSO_4_·5H_2_O, 0.05 mmol L^−1^ potassium ferricyanide) at 37°C for 150 min. The reagents were purchased from WAKO pure chemicals (Tokyo, Japan). Eserine‐hemisulfate salt was added to the assay medium at a concentration of 10 mmol L^−1^ in order to inhibit AChE activity. Slides were then thoroughly washed with 50 mmol L^−1^ Tris‐HCl (Tris) buffer (pH 7.6) for 3 min with five changes and then incubated for 5 min with a solution containing 0.04% 3,3′‐diaminobenzidine 4HCl and 0.3% nickel–ammonium in 50 mmol L^−1^ Tris buffer (pH 7.6). Subsequently, 0.3% H_2_O_2_ solution was added at a final concentration of 0.003% to the assay solution. After standing at room temperature for 10 min, slides were finally washed with 50 mmol L^−1^ Tris buffer.

In the colorimetric enzyme assay: The isolated ileal mucosa and muscle layer which was approximately 1 cm in length, was placed in a microtube and allowed to stand for 60 min on ice in order to oxidize endogenous free SH group residues with oxygenated Krebs‐Ringer buffer solution. The tubes were then centrifuged. The precipitated tissues were weighed after washing once with ice‐cold 0.1 mol L^−1^ phosphate buffer (pH 7.3) and stored at −80°C. The precipitated tissues were thawed, cut on ice, and crushed at 25 sec^−1^ with a ninefold volume of 1% Triton X100 in 0.1 mol L^−1^ phosphate buffer (pH 7.3) by the Tissue lyser (Qiagen, Hilden, Germany). The supernatant was then isolated as the crude tissue extract after centrifugation of the tissue lysate (21,000 ***g***, 30 min at 4°C). Protein concentrations were measured by the Quick Start protein assay using Bradford Dye reagent (Bio‐Rad Laboratories; CA) with bovine serum albumin as a protein standard. The colorimetric assay of AChE was measured using 1‐methyl‐4‐acetylthromethilpiperidine (MATP^+^; DOJINDO Lab, Kumamoto, Japan) for a selective AChE assay by the method of Kikuchi et al. (2010). Human erythrocyte AChE (SIGMA‐Aldrich Japan, Tokyo, Japan; hAChE) was used as a standard for activity in each assay experiment in order to convert crude enzyme activities to the specific activities of hAChE (munits mg^−1^ of protein min^−1^). Enzyme activity was monitored at 415 nm with reference to 595 nm by a kinetic analysis using a microplate reader (BioRAD model 680, Tokyo, Japan).

### Real‐time quantitative reverse transcriptase PCR (RT‐PCR) in epithelial cells

Total RNA was prepared using TRIzol (Life Technologies) from isolated epithelia. First‐strand cDNA synthesis was completed using ReverTra Ace (TOYOBO, Osaka, Japan). Quantitative PCR reactions were conducted with Rotor Gene 6000 equipment (Qiagen, Hilden, Germany) using the KAPA SYBR Green Fast PCR kit (KAPA Biosystems, Woburn, MA). The specific primers used for the quantitative RT‐PCR analysis of epithelial cells from the ileum were as follows: GPR43 (NM_146187.3); mGPR43‐F_qpcr: TCCTAGACCCAGTGACTGGTGAC; mGPR43‐R_qpcr: TTTGTACATGTGCTCCGCTGA, choline transporter‐like protein 4 (CTL4) (NM_023557); mSlc44a4‐F_qpcr: AGAATGAGAACGAGGCTCACG; mSlc44a4‐R_qpcr: CCCCACTATGATGTAACCCAAAA, Chat (NM_009891); mChat‐F_qpcr: GGCCATTGTGAAGCGGTTTG: mChat‐R_qpcr:GCCAGGCGGTTGTTTAGATACA: AChE (NM_001290010); mAChE‐F_qpcr: CTCCCTGGTATCCCCTGCATA; mAChE‐R_qpcr: GGATGCCCAGAAAAGCTGAGA: Gapdh; RT2 qPCR Primer Assay for Mouse GAPDH (Qiagen, Venlo, the Netherlands, Cat. No. PPM02946E).

### Statistical analyses

Differences among experimental groups were analyzed using a one‐way ANOVA following by a post hoc test using Scheffe for statistical analyses and the *χ* ^2^ method to compare the appearance rate of contractile responses to SCFAs. Differences among groups were considered significant at *P *<* *0.05. Data are expressed as the mean ± SEM.

## Results

### Ileal contractile responses to acetate and propionate in neonatal and adult mice

**Figure 1:**
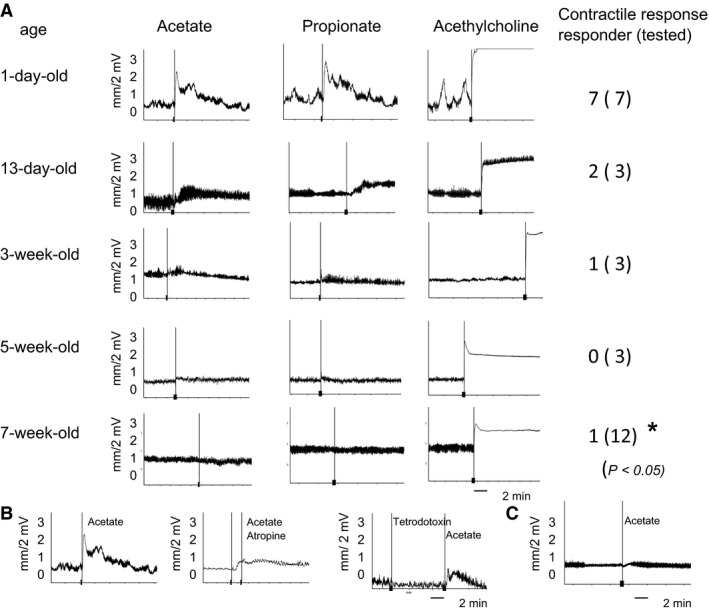
Chart of ileal contractile responses to acetate and propionate. Ileal contractions were measured in 1‐day‐old, 13‐day‐old, 3‐week‐old, 5‐week‐old, and 7‐week‐old animals using the Magnus isotonic‐transducing system. (A) Charts show contractile responses to sodium acetate (2.5 mmol L^−1^), sodium propionate (1 mmol L^−1^), and acetylcholine chloride (1 *μ*mol L^−1^). Ileal contractions occurred in all 1‐day‐old pups, and were desensitized in 7‐week‐old mice (*P *<* *0.05). The number of responders is shown and that of tested animals are shown in parenthetic numbers. (B) Inhibitory effects on ileal contractile responses to SCFA. Inhibition tests were performed in order to determine whether SCFA‐sensing contractions followed the production of ACh. Atropine (5 *μ*mol L^−1^), a blocker of muscarinic acetylcholine receptors, completely inhibited ileal contractions to acetate in 1‐day‐old pups. SCFA‐sensing contractions were not inhibited by a pretreatment with 10^−6^ mol L^−1^ tetrodotoxin. (c) The response induced by 2.5 mmol L^−1^ acetate was absent in the mucosa‐free ileum of 1‐day‐old pups.

We examined ileal contractile responses to SCFAs from 1‐day‐old pups to 7‐week‐old mice. Contractile responses to acetate or propionate were observed in all 1‐day‐old pups (Fig. 1A). These responses were desensitized in 5‐week‐old and 7‐week‐old mice; the appearance rate of the responders in 7‐week‐old mice was 8% (1/12) (Fig. 1A). The *χ* ^2^ method analysis confirmed that the appearance rate of ileal contractions was significantly lower in adult mice than in 1‐day‐old pups. This result revealed that ileal contractile responses to acetate or propionate decreased with age, and were almost desensitized in adult mice. The treatment with ACh induced ileal contractions in all mice tested (Fig. 1A).

Inhibition tests were then performed in order to determine the involvement of ACh in SCFA‐sensing contractions. Atropine, a blocker of muscarinic ACh receptors, strongly inhibited ileal contractions to acetate in all 1‐day‐old pups (Fig. 1B), suggesting that ACh plays a role in these contractile responses. The pretreatment with tetrodotoxin did not inhibit contractile responses to acetate (Fig. 1B). Since ileal contractile responses to ACh occurred in nonresponding adult mice (Fig. 1A), desensitized contractile responses may be attributed to reductions in the release of ACh. When the ileal mucosal layer in 1‐day‐old pups was scraped off, no contractile responses to acetate were observed (Fig. 1C), indicating that these responses require the mucosal layer in order to sense SCFAs and release ACh.

### Ileal AChE activities

**Figure 2:**
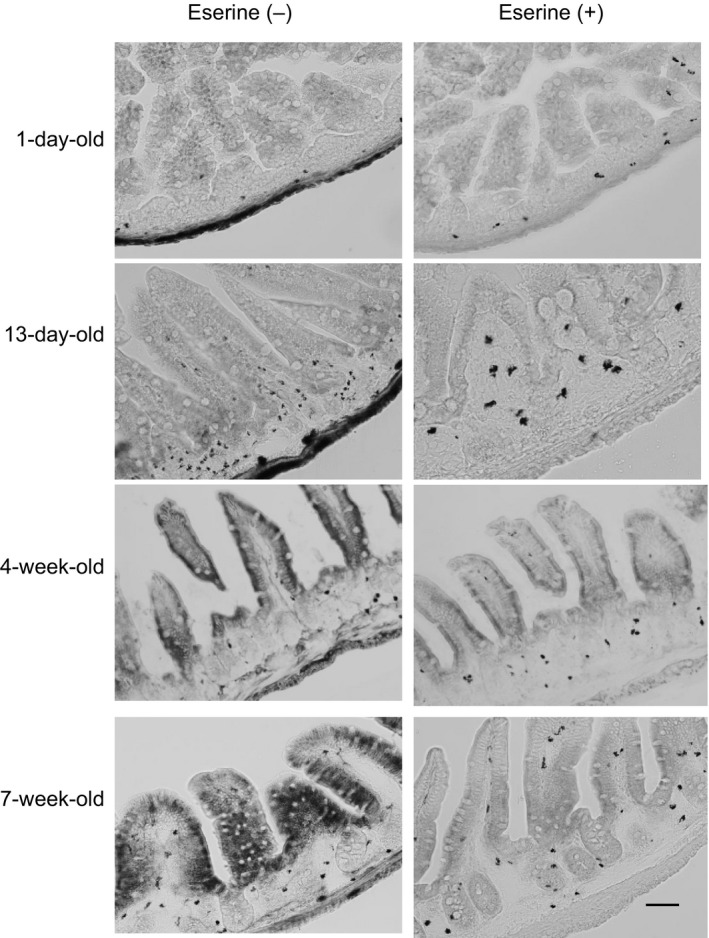
Developmental expression of acetylcholinesterase (AChE) activities in the terminal ileum. AChE activities were determined by histochemical analyses in mice. A histochemical analysis was performed using the modified method of Karnovsky and Roots in 1‐day‐old, 13‐day‐old, 4‐week‐old, and 7‐week‐old Conv without (no treatment) and with eserine (an inhibitor of AChE), respectively. AChE activity was absent in the ileal villi of neonates, but was strongly detected in those of adults; however, age‐dependent differences were not noted in muscular activity. Scale bar; 100 *μ*m.

In order to determine the role of the decreased release of ACh, we histochemically examined AChE activities in the ileum of mice at various ages. As shown in Figure 2, AChE activities were almost absent in the mucosal epithelium of 1‐day‐old and 13‐day‐old pups, but were clearly detected in the myenteric and submucosal plexuses, similar to adult mice. In contrast, the epithelia of 4‐week‐old and 7‐week‐old mice exhibited strong AChE activities and an AChE‐positive nerve fiber‐like structure was detected in the ileal lamina propria. Positive reactions for AChE activities were not detected in the presence of the AChE inhibitor, eserine. This result suggests that epithelial AChE activity gradually increases in intensity with development in order to inhibit the release of epithelial ACh in the adult stage.

**Figure 3:**
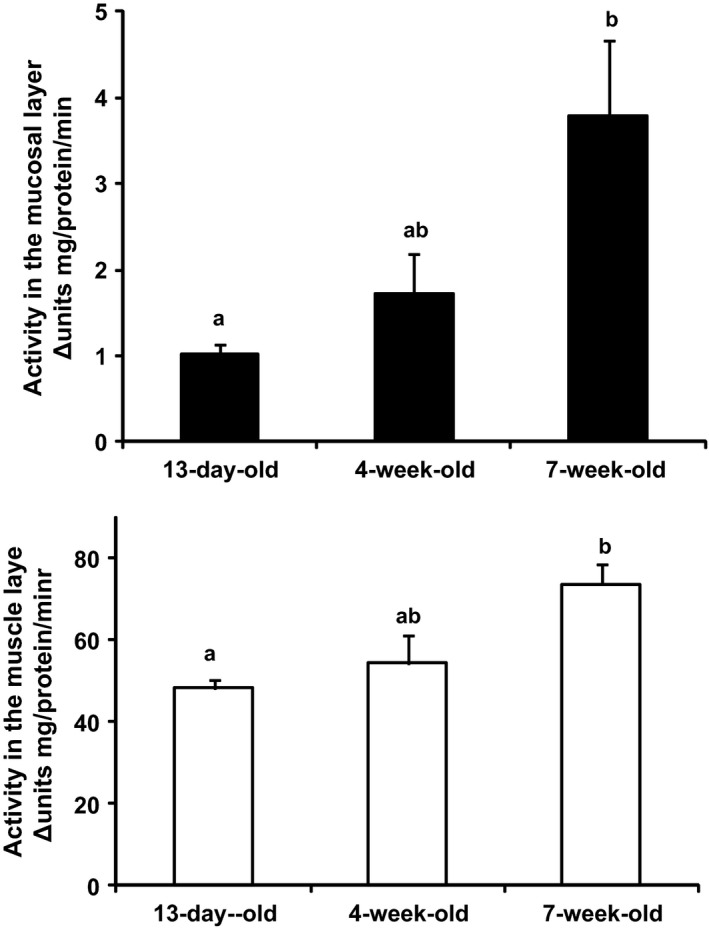
Colorimetric analysis of age‐dependent activities of acetylcholinesterase (AChE) in the terminal ileum of mice. The colorimetric assay of AChE was performed using 1‐methyl‐4‐acetylthromethilpiperidine (MATP ^+^; DOJINDO Lab, Kumamoto, Japan) for a selective AChE assay by the method of Kikuchi et al. ^S2^ Human erythrocyte AChE (SIGMA‐Aldrich Japan, Tokyo, Japan; hAChE) was used for standard activity in each assay in order to convert crude enzyme activities to comparable specific activities of hAChE (munits min^−1^ mg^−1^ of protein mL^−1^). The colorimetric analysis showed that the activities of the crude tissue extracts of villous mucosa and mucosa‐free ileal tissues were significantly higher in 7‐week‐old than in 13‐day‐old animals. Data are shown as the mean ± SEM of *n* = 4 mice per group. The different letters that are shown at each error bar indicate significant differences (*P *<* *0.05) by a one‐way ANOVA.

In an attempt to quantitatively evaluate the AChE activity, we separated the mucosal layer from the muscular layer and measured enzyme activities using colorimetric analysis. As shown in Figure 3, mucosal AChE activity in the ileum was significantly lower (3.5‐fold) in 13‐day‐old pups than in 7‐week‐old mice (*P* = 0.0323). Meanwhile, muscular AChE activity was also significantly lower in 13‐day‐old pups than in 7‐week‐old mice (*P* = 0.0148), but was 20–50‐fold higher than that in the mucosal layer at each age. The analysis of AChE activity in crude tissue extracts revealed the same age‐dependent increases, which were consistent with the histochemical findings. Therefore, the increase observed in AChE activities after weaning may decrease the release of ACh due to the desensitization of SCFA‐sensing contractions.

### An inhibitor of AChE restored desensitized contractile responses

**Figure 4:**
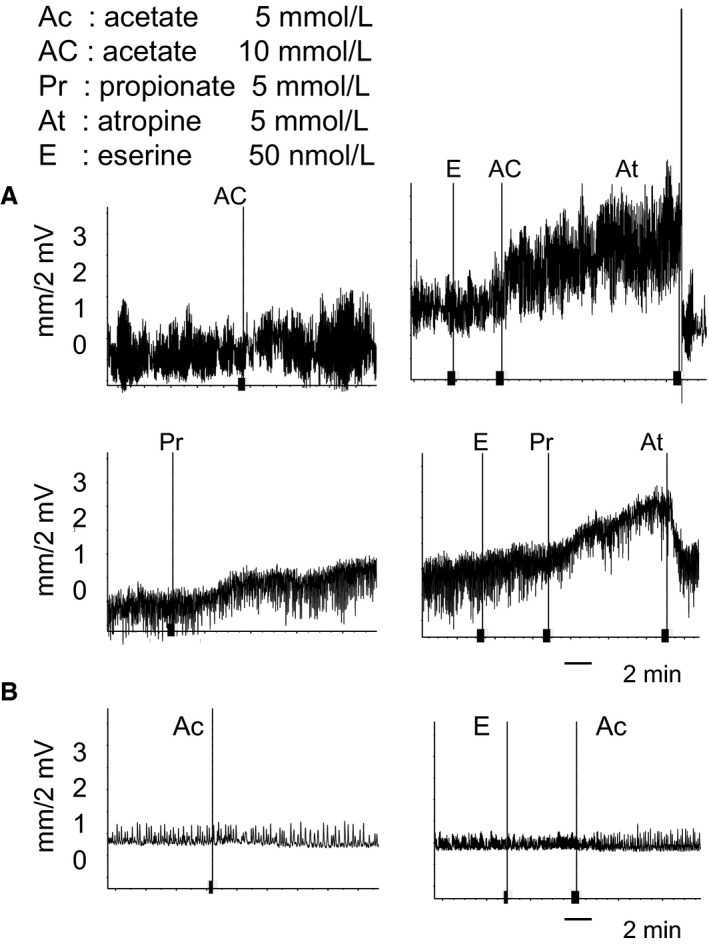
An inhibitor of AChE, eserine, restored desensitized responses in adult Conv. (A) Desensitized ileal contractions to acetate and propionate were restored by the 2‐min prior addition of 50 nmol L^−1^ eserine and were completely inhibited by the addition of atropine (*n* = 4). (B) The restoration of contractions to acetate by the 2‐min prior addition of 50 nmol L^−1^ eserine was not induced in a mucosa‐free ileum from which the mucosal layer had been scraped off.

We subsequently determined whether eserine, an inhibiter of AChE, had the ability to restore ileal contractile responses in adult mice. Desensitized ileal contractions in response to acetate or propionate were recovered by the 2‐min prior addition of eserine and were completely inhibited by atropine at each time point (Fig. 4A). The recovery of ileal contractile responses by the pretreatment with eserine did not occur when the mucosal layer was scraped off (Fig. 4B). These results indicate that AChE actively hydrolyzes released ACh in order to desensitize ileal contractile responses to SCFAs in the ileum of adult mice.

### Ileal contractile responses in adult animals with systemic inflammation

**Figure 5:**
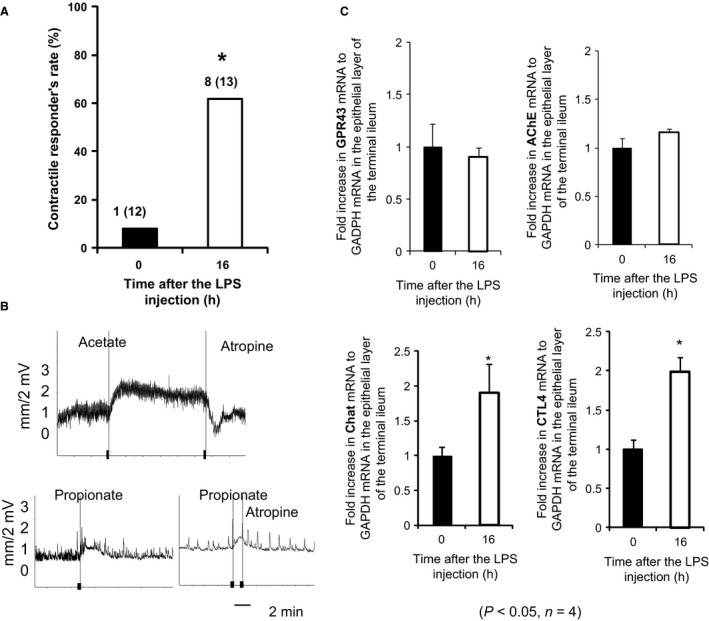
Effects of an intraperitoneal injection of LPS on SCFA‐sensing ileal contractile responses and their appearance rate, and mRNA expression with acetylcholine synthesis, transport, and degradation in the epithelial layer of 7‐week‐old mice. Ileal contractile responses were measured in adult mice 16 hrs after a peritoneal injection of LPS using the Magnus isotonic‐transducing system. (A) Comparison of the appearance rate (%) of ileal contractile responses in 7‐week‐old mice without (■) and with (□) an intraperitoneal injection of LPS. The numbers of responders to SCFAs and tested animals (responder/tested) were shown at the top of each column. The recovered appearance rate increased significantly with LPS (*P *<* *0.05 by *χ* ^2^ analysis). (B) Contractions to acetate or propionate were completely inhibited after the addition of 5 *μ*mol L^−1^ atropine. (C) Comparison of the mRNA expression levels of GPR43, acetylcholinesterase (AChE), choline acetyltransferase (Chat), and choline transporter‐like protein 4 (CTL4) in the epithelial layer of 7‐week‐old mice with or without an intraperitoneal injection of LPS. The gene expression levels of Chat and CTL4 increased significantly (twofold) with LPS, while those of GPR43 and AChE did not change. Data are shown as the mean ± SEM (*P <* *0.05) of *n* = 4 mice.

A previous study reported that the mRNA expression of AChE and its activity decreased in the splenocytes and serum of mice 24 h after an intraperitoneal injection of LPS (Shaked et al. 2009). Therefore, we herein investigated whether an injection of LPS into the peritoneal cavity of 7‐week‐old mice affected the appearance rate of contractile responses to SCFAs. As shown in Figure 5A, contractions appeared in response to 10 mmol L^−1^ acetate and 5 mmol L^−1^ propionate 16 h after the LPS injection in 7‐week‐old mice, but did not occur in response to the same dose of acetate or propionate without the LPS injection (already shown in Fig. 4B). The appearance rate (%) of contractile responses was significantly higher with (62%) than without (8%) the LPS injection (Fig. 5A). The restored contractions to acetate and propionate in LPS‐treated mice were completely inhibited after the addition of atropine (Fig. 5B). This result suggests that the release of ACh is involved in the induction of SCFA‐sensing contractions by LPS.

### mRNA expression of GPR43 and ACh‐related enzymes

As described above (Fig. 1C), the mucosal layer is required to induce ileal contractile responses to SCFAs. Therefore, using an isolated ileal epithelium, we attempted to identify the factors involved in the recovery of contractile responses after an LPS injection. When we examined the mRNA expression levels of GPR43 as a SCFA receptor in the epithelial layer of 7‐week‐old mice, no significant differences were observed in the expression of GPR43 with and without LPS (Fig. 5C). We then measured gene expression levels related to the release of ACh: Chat, CTL4, and AChE, which are required for the synthesis, secretion, and hydrolyzation of ACh, in the ileal epithelium of 7‐week‐old mice. The mRNA expression levels of Chat and CTL4 were significantly higher (twofold) 16 h after the LPS injection, whereas that of AChE did not change (Fig. 5C). These results indicate that LPS may enhance the synthesis and secretion of ACh more than ACh hydrolyzation activity.

## Discussion

Although luminal SCFAs predominantly accumulate in the large intestine, sufficient amounts of SCFAs have also been detected in the terminal ileum (Dass et al. 2007). In this study, we targeted the ileum, but not the colon in order to examine contractile responses to SCFAs because the colon of neonatal mice is too short to separate into three functional segments (proximal, middle, and distal colon) (Yajima 1985) for mounting in the Magnus chamber. We confirmed in a preliminary experiment that the terminal ileum sensed SCFAs, resulting in strong contractile responses similar to those in the colon of 1‐day‐old pups. We herein demonstrated for the first time that non‐neuronal, but atropine‐sensitive contractile responses to acetate or propionate occurred in the ileum; however, they became desensitized with age. Desensitized responses were restored under the inflammatory conditions induced by LPS. These ileal contractile responses to SCFAs did not occur in the absence of the mucosal layer and were not inhibited by tetrodotoxin (Fig. 1), indicating that epithelial non‐neuronal ACh is involved in SCFA‐sensing ileal contractions. We also demonstrated that the recall of contractions under inflammatory conditions from desensitized responses may be caused by cholinergic alterations.

Regarding the mechanism underlying the desensitization of SCFA‐sensing ileal contraction in adult mice, we showed for the first time that the developmental up‐regulation of mucosal AChE activity around the weaning period contributed to the induction of desensitization, possibly via a decrease in the concentration of ACh released. We additionally noted that ChAT mRNA expression levels in the whole tissue of the ileum decreased in an age‐dependent manner to approximately 20% in 7‐week‐old mice (Figure S1A). The age‐dependent decreases observed in Chat mRNA levels may partially contribute to the appearance of the desensitization of atropine‐sensitive contractions.

At the point of developmental increases in AChE activity after birth, we detected an alteration in AChE activity not only in the mucosa (Fig. 3A) but also in the muscular site (Fig. 3B) in the ileum. Muscular AChE activity, which is mainly derived from muscular cholinergic neurons, also increased in an age‐dependent manner. Furthermore, we detected AChE‐positive nerve fiber‐like structures in the ileal lamina propria in mice older than 4 weeks, but not in the neonatal period using the histochemical test for AChE activity (Fig. 2). Regarding developmental increases in neuronal AChE activity, Roberts et al. (2007) previously reported that AChE‐positive neuron‐mediated spontaneous motility patterns were first observed in the colon of 6‐day‐old mice and the adult pattern appeared by 10 days. Another study showed that histochemical AChE activity in the myenteric plexuses of the colon decreased by 70% in toll‐like receptor (TLR) 2 knockout mice (Wang et al. 2010; Brun et al. 2013). Collectively, these findings and the present results indicate that the up‐regulation of neuronal‐ and non‐neuronal‐AChE activities is accelerated in accordance with the commencement of bacterial habitation in the intestine after birth.

We also found that once‐desensitized contractions were restored under LPS‐induced inflammatory conditions. AChE activities in the splenocytes and serum of mice were previously reported to be significantly lower under LPS‐induced inflammatory conditions (Shaked et al. 2009). In contrast to these findings, the expression levels of ileal‐epithelial AChE in this study did not change following an intraperitoneal injection of LPS (Fig. 5C). However, the inhibitory experiments performed herein revealed that restored contractions under LPS‐induced inflammatory condition were sensitive to atropine (Fig. 5B); therefore, these contractions may involve the release of non‐neuronal ACh. Furthermore, the epithelial gene expression of Chat and CTL4 was significantly increased after the LPS injection (Fig. 5C). A recent study showed that CTL4 played a key role in the synthesis and release of ACh in non‐neuronal cells (Song et al. 2013). We noted that the expression of CTL4 gradually increased with age in the epithelial layers in mice (Figure S2). Therefore, CTL4 and Chat may contribute to enhancing the release of epithelial non‐neuronal ACh in LPS‐induced inflammation.

GPR43 is known to be expressed in intestinal epithelial cells and senses SCFAs in humans (Le Poul et al. 2003; Karaki et al. 2008), rats (Karaki et al. 2006), and mice (Kim et al. 2013). Regarding the involvement of GPR43 in intestinal contractions to sense SCFAs, Dass et al. (2007) reported inconsistent data for colonic circular muscle contractions in rats and mice using an electrical field stimulation (EFS). SCFAs inhibited colonic contractions post‐FES, and contractions were completely inhibited by 10^‐6^M tetrodotoxin. Furthermore, the inhibitory effects of propionate were similar in tissues with or without mucosa, and in wild‐type and GPR43 gene knockout mice. They concluded that the inhibitory effects of SCFA may be independent of the GPR43 receptor. However, the contractile responses to SCFAs in our experiments were completely different from their findings. They detected neuronally activated contractions in circular muscle, whereas we observed non‐neuronally induced contractions in the longitudinal muscle of the ileum, which may have been induced by the release of ACh following the sensing of SCFAs and were not inhibited by 10^‐6^M tetrodotoxin. Since non‐neuronal ACh‐induced ileal contractions did not appear without a mucosal layer in neonates (Fig. 1C) or LPS‐inflammatory adult mice (Fig. 4B), any epithelial SCFA receptors may be involved in sensing acetate or propionate following the epithelial release of ACh. The G protein, G*α*_q/11_, has been identified as a key factor in the function of GPR43 (Le Poul et al. 2003). In the rat colon, the non‐neuronal release of ACh induced by propionate was shown to be completely inhibited by YM‐254890, a selective inhibitor of G*α*_q/11_ (Yajima et al. 2011). Therefore, GPR43 is potentially involved in SCFA sensing and also atropine‐sensitive ileal contractions, which were found to be mediated by non‐neuronal ACh in mice. The involvement of GPR43 in the sensing of SCFAs in atropine‐sensitive ileal contractions is consistent with its unchanged expression in the ileal epithelium under LPS‐induced inflammation (Fig. 5C). GPR43 may be involved in desensitizing contractile changes in response to acetate or propionate in adult mice because the expression levels of GPR43 mRNA in the whole tissue of the terminal ileum decreased with age (Figure S1B).

We herein provided evidence for the first time for a physiological role for ileal non‐neuronal ACh, which contributes to the maintenance of homeostatic functions by regulating non‐neuronal ileal SCFA‐sensing contractions in health and disease. The desensitization of ileal contractions to SCFAs in healthy adults was associated with developmental increases in epithelial AChE, which may contribute to the avoidance of excessive responses to the luminal accumulation of SCFAs. However, the restoration of desensitized atropine‐sensitive contractions in adult mice after the LPS injection appeared to be regulated by a balance shift between the production and hydrolysis of epithelial ACh. The accelerated release of epithelial ACh by the sensing of SCFAs under inflammatory conditions may additionally help to wash away the intestinal contents by subsequent ileal contractions, and also supply ACh for cholinergic anti‐inflammatory actions through nicotinic acetylcholine receptor *α*7 (Wang et al. 2003). Ileal contractile responses to SCFAs under health and inflammatory conditions may contribute to the epithelial release of non‐neuronal ACh in order to sustain homeostasis in a host in spite of disturbances by opportunistic or pathological infections with inflammation in intestinal disorders.

In conclusion, non‐neuronal and atropine‐sensitive contractions to SCFAs constitutively occurred in the ileum of neonatal mice, and were age‐dependently desensitized, but restored after an intraperitoneal injection of LPS into adult mice. We demonstrated for the first time that non‐neuronal ACh and SCFAs may coincidentally play a role in the regulation of ileal contractile responses under healthy and inflammatory conditions.

## Conflicts of Interest

None declared.

## Supporting information




**Figure S1.** Age‐dependent changes in ileal mRNA expression levels in mice; choline‐acetyl transporter (Chat) and short‐chain fatty acid receptor GPR43. The relative expression level of mRNA was calculated using the comparative Ct method by subtracting the Ct value of *β* actin mRNA from that of the target mRNA. (A) Changes in the mRNA expression levels of (A) Chat and (B) GPR43 in the ileum of 1‐day‐old, 15‐day‐old, 3‐week‐old, 5‐week‐old, and 7‐week‐old animals (*n* = 3–4, *P *<* *0.05). Expression levels gradually decreased with age to 20% that of 1‐day‐old pups.
**Figure S2.** In situ hybridization of choline transporter‐like protein 4 (CTL4) mRNA. Two non‐overlapping antisense oligonucleotide DNA probes were designed for the mRNA of mouse choline transporter‐like protein 4 (CTL4). These probes were labeled with ^33^P‐dATP using terminal deoxynucleotidyl transferase. Hybridization was performed at 42°C for 10 h with a hybridization buffer containing ^33^P‐labeled oligonucleotide probes (10,000 cpm *μ*L^−1^). The hybridized sections were dipped in an autoradiographic emulsion (NTB‐2; Kodak) at 4°C for 8–10 weeks. CTL4 mRNA was not detected in the ileum of 15‐day‐old pups (the left panel), but was strongly detected in the epithelial layers of the terminal ileum of adult mice (the right panel).
