# Supplementary figures and images for: Non‐neuronal, but atropine‐sensitive ileal contractile responses to short‐chain fatty acids: age‐dependent desensitization and restoration under inflammatory conditions in mice
Source: Physiol Rep. 2016 Apr 6;4(7):e12759. doi: 10.14814/phy2.12759 (PMC4831327; doi:10.14814/phy2.12759)

## Supporting Figure 1

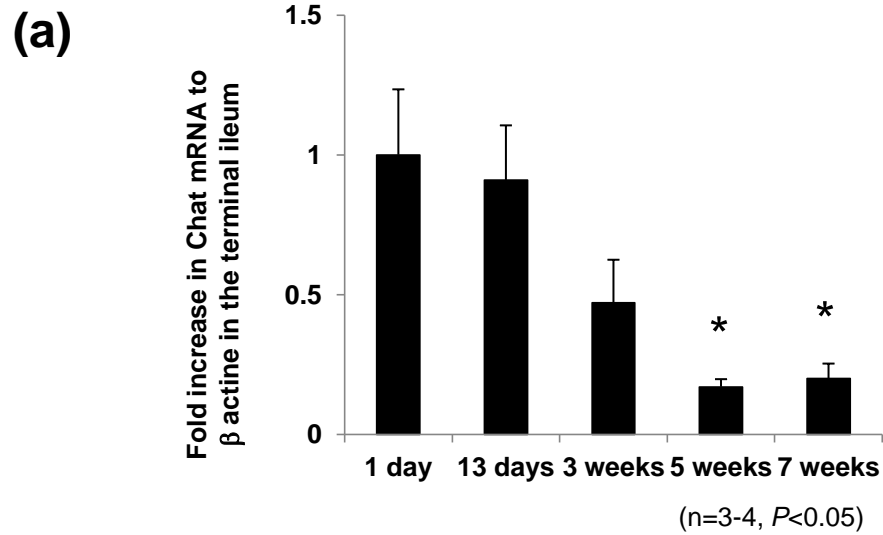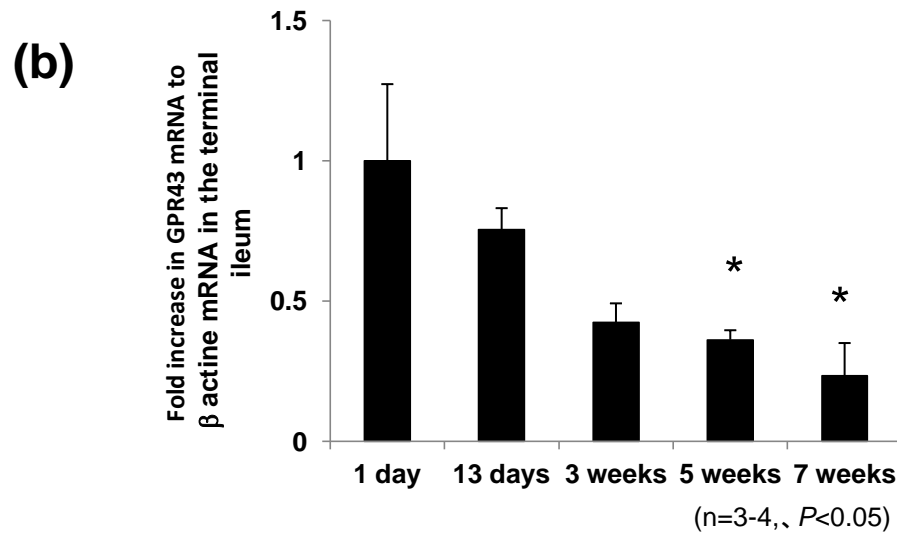

## Supporting Figure 2

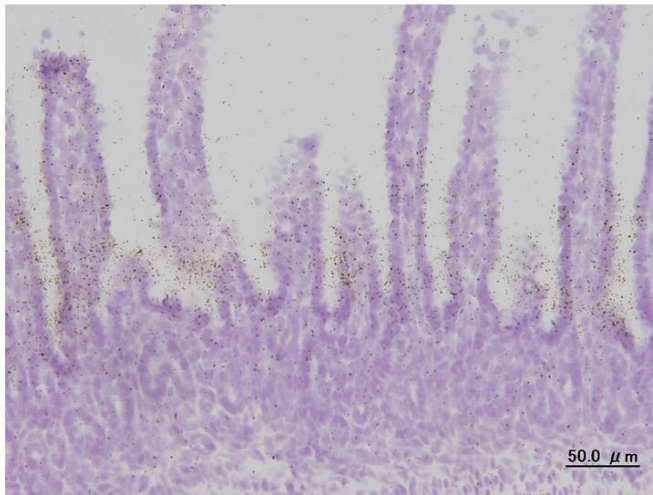

day 15

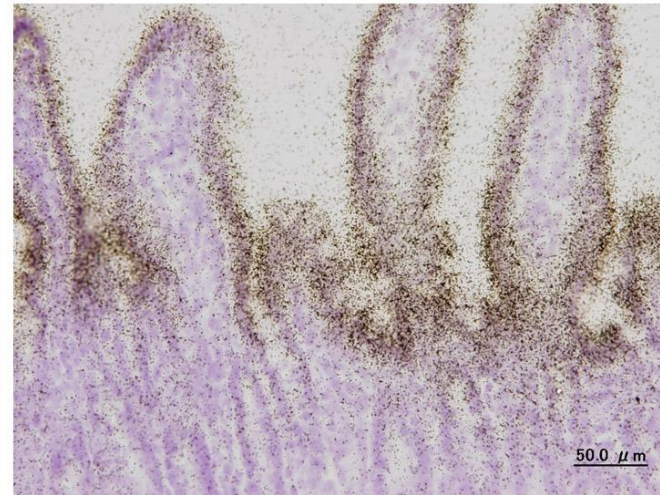

adult

Supplement: Supplementary file 1 — Figure S1. Age‐dependent changes in ileal mRNA expression levels in mice; choline‐acetyl transporter (Chat) and short‐chain fatty acid receptor GPR43. The relative expression level of mRNA was calculated using the comparative Ct method by subtracting the Ct value of β actin mRNA from that of the target mRNA. (A) Changes in the mRNA expression levels of (A) Chat and (B) GPR43 in the ileum of 1‐day‐old, 15‐day‐old, 3‐week‐old, 5‐week‐old, and 7‐week‐old animals (n = 3–4, P < 0.05). Expression levels gradually decreased with age to 20% that of 1‐day‐old pups. Figure S2. In situ hybridization of choline transporter‐like protein 4 (CTL4) mRNA. Two non‐overlapping antisense oligonucleotide DNA probes were designed for the mRNA of mouse choline transporter‐like protein 4 (CTL4). These probes were labeled with 33P‐dATP using terminal deoxynucleotidyl transferase. Hybridization was performed at 42°C for 10 h with a hybridization buffer containing 33P‐labeled oligonucleotide probes (10,000 cpm μL−1). The hybridized sections were dipped in an autoradiographic emulsion (NTB‐2; Kodak) at 4°C for 8–10 weeks. CTL4 mRNA was not detected in the ileum of 15‐day‐old pups (the left panel), but was strongly detected in the epithelial layers of the terminal ileum of adult mice (the right panel). [file PHY2-4-e12759-s001.pdf]
