# Supplementary material for: Non‐neuronal, but atropine‐sensitive ileal contractile responses to short‐chain fatty acids: age‐dependent desensitization and restoration under inflammatory conditions in mice
Source: Physiol Rep. 2016 Apr 6;4(7):e12759. doi: 10.14814/phy2.12759 (PMC4831327; doi:10.14814/phy2.12759)
Supplement: Supplementary file 2 [file PHY2-4-e12759-s002.docx]

**Supporting Information**

**Supporting Methods**

*Real-time quantitative reverse transcriptase PCR (RT-PCR) in the whole tissue of the ileum*

Mice were asphyxiated with CO_2_ gas and immediately exsanguinated following bloodletting. Ileal segments were immediately placed in Allprotect reagent (QIAGEN, Tokyo, Japan) and stored at -80 °C until their use following overnight refrigeration. Total RNA was purified using QuickGene RNA tissue kit S (FUJIFILM, Tokyo, Japan) and the RNase-free DNase set (TaKaRa, Shiga, Japan) with the nucleic acid isolation system QuickGene-810 (FUJIFILM, Tokyo, Japan) according to the manufacturer’s instructions. The synthesis of cDNA (240 ng of total RNA per 20 μL reaction mixture) was performed using the Prime Script RT reagent kit (Perfect Real Time) RR037A (TaKaRa) according to the manufacturer’s instructions. Real-time quantitative PCR was performed using ABI 7300 (Life Technologies, Carlsbad, CA, USA). The expression of the housekeeping gene, β actin, was assessed as a control. In our preliminary study, the relative expression level of the other housekeeping gene, glyceraldehyde-3-phosphate dehydrogenase (GAPDH), was approximately equal to that of β actin in the ileum. TaqMan primer and probe sets for each gene (choline acetyltransferase; NM_009891: GPR43; NM_146187.3: β actin; NM_007393) were purchased from Biosearch Technologies Japan (Tokyo, Japan). All reactions were conducted with Premix Ex Taq Perfect Real Time RR039A (TaKaRa) according to the manufacturer’s instructions. The relative expression level of mRNA was calculated using the comparative Ct method by subtracting the Ct value of β actin mRNA from the Ct value of the target mRNA.

In situ *hybridization of choline transporter-like protein 4 in the ileum*

Two non-overlapping antisense oligonucleotide DNA probes were designed for the mRNA of mouse choline transporter-like protein 4 (CTL4): 841–885 and 1501–1545 of CTL4 mRNA (accession number: NM_023557). These probes were labeled with ^33^P-dATP using terminal deoxynucleotidyl transferase (Invitrogen, Carlsbad, CA). Freshly frozen 14-μm-thick sections were fixed with 4% paraformaldehyde in 0.1 M phosphate buffer for 15 min and then acetylated with 0.25% acetic anhydride in 0.1 M triethanolamine-HCl (pH 8.0) for 10 min. Hybridization was performed at 42°C for 10 hrs with a hybridization buffer containing ^33^P-labeled oligonucleotide probes (10,000 cpm μL^-1^). The hybridized sections were dipped in an autoradiographic emulsion (NTB-2; Kodak) at 4°C for 8-10 weeks.

**Supporting Figure Legends**

Supporting Figure 1. Age-dependent changes in ileal mRNA expression levels in mice; choline-acetyl transporter (Chat) and short-chain fatty acid receptor GPR43. The relative expression level of mRNA was calculated using the comparative Ct method by subtracting the Ct value of β actin mRNA from that of the target mRNA. (a) Changes in the mRNA expression levels of (a) Chat and (b) GPR43 in the ileum of 1-day-old, 15-day-old, 3-week-old, 5-week-old, and 7-week-old animals (n=3-4, *P*<0.05). Expression levels gradually decreased with age to 20% that of 1-day-old pups.

Supporting Figure 2. *In situ* hybridization of choline transporter-like protein 4 (CTL4) mRNA. Two non-overlapping antisense oligonucleotide DNA probes were designed for the mRNA of mouse choline transporter-like protein 4 (CTL4). These probes were labeled with ^33^P-dATP using terminal deoxynucleotidyl transferase. Hybridization was performed at 42°C for 10 hrs with a hybridization buffer containing ^33^P-labeled oligonucleotide probes (10,000 cpm μL^-1^). The hybridized sections were dipped in an autoradiographic emulsion (NTB-2; Kodak) at 4°C for 8-10 weeks. CTL4 mRNA was not detected in the ileum of 15-day-old pups (the left panel), but was strongly detected in the epithelial layers of the terminal ileum of adult mice (the right panel).
